# Supplementary material for: A Quantum Mechanical MP2 Study of the Electronic Effect of Nonplanarity on the Carbon Pyramidalization of Fullerene C60
Source: Nanomaterials (Basel). 2024 Sep 29;14(19):1576. doi: 10.3390/nano14191576 (PMC11477707; doi:10.3390/nano14191576)
Supplement: Supplementary file 1 [file nanomaterials-14-01576-s001.zip › nanomaterials-3154788-supplementary.pdf]

Table S1. The cartesian coordinates of optimized structure for fullerene C_{60} (MP2/6-31G*).

Atom	X	Y	Z
C	12.105033	20.482125	-8.339266
C	13.552033	20.482163	-8.339324
C	14.255421	21.700532	-8.339355
C	13.531886	22.953654	-8.339352
C	12.125054	22.953619	-8.339295
C	11.401586	21.700460	-8.339257
C	11.657883	19.388517	-9.174666
C	12.828529	18.712663	-9.691020
C	13.999181	19.388572	-9.174744
C	15.137294	19.543719	-9.987028
C	15.426029	21.860103	-9.174834
C	14.255335	23.887699	-9.174810
C	13.551865	24.795772	-9.987036
C	12.104867	24.795735	-9.986984
C	11.401498	23.887627	-9.174709
C	10.230857	23.211711	-9.690988
C	10.230900	21.859972	-9.174641
C	9.796168	20.796723	-9.986851
C	10.519696	19.543599	-9.986860
C	12.828496	18.210659	-11.005237
C	11.657810	18.370165	-11.840624
C	10.519666	19.027265	-11.338603
C	9.796107	19.961267	-12.174015
C	9.348954	21.054853	-11.338585
C	9.348918	22.369070	-11.840591
C	9.796054	23.462685	-11.005193
C	10.519511	24.396722	-11.840658
C	11.657660	25.053875	-11.338718
C	15.425982	23.211845	-9.691169
C	13.998888	24.035586	-14.004761
C	15.137075	23.880502	-13.192567
C	15.860603	22.627379	-13.192575
C	15.425872	21.564130	-14.004786
C	14.255185	21.723642	-14.840170
C	12.104739	22.941939	-14.840103
C	11.657590	24.035529	-14.004681
C	12.828242	24.711440	-13.488408
C	12.828275	25.213442	-12.174190
C	13.998960	25.053936	-11.338803
C	15.137105	24.396837	-11.840823
C	16.307818	22.369249	-11.840842
C	16.307853	21.055032	-11.338836
C	15.860717	19.961417	-12.174233

C	15.425915	20.212391	-13.488439
C	14.255273	19.536476	-14.004718
C	13.531717	20.470483	-14.840131
C	12.124885	20.470448	-14.840076
C	11.401351	21.723570	-14.840071
C	10.519475	23.880385	-13.192399
C	9.795999	22.627229	-13.192361
C	10.230742	21.563999	-14.004592
C	10.230790	20.212257	-13.488257
C	11.401435	19.536403	-14.004617
C	12.104905	18.628330	-13.192389
C	13.551904	18.628367	-13.192443
C	13.999110	18.370228	-11.840709
C	15.137261	19.027379	-11.338768
C	15.860663	23.462834	-11.005412
C	15.860772	20.796873	-9.987066
C	13.551739	22.941978	-14.840160
